# Isolated TERT promoter mutation may define a high-risk prognostic subgroup in papillary thyroid carcinoma: an exploratory, hypothesis-generating analysis of TCGA and GSE33630 datasets

**DOI:** 10.3389/fendo.2026.1881969

**Published:** 2026-10-15

**Authors:** Bing Guan, Rong Zhou, Sijia Zhang, Yawen Chen, Lulu Li, Ping He

**Affiliations:** Department of Pathology, Suzhou BenQ Medical Center, Suzhou, Jiangsu, China

**Keywords:** BRAF V600E, papillary thyroid carcinoma, prognosis, progression-free interval, TCGA, TERT promoter mutation, transcriptomics

## Abstract

**Background:**

The prognostic value of combined BRAF V600E and TERT promoter (TERTp) mutations in papillary thyroid carcinoma (PTC) has been established in surgical cohorts. However, the independent impact of TERTp mutation in the absence of BRAF mutation remains underexplored, particularly within publicly available genomic datasets.

**Methods:**

We analyzed 49 PTC patients with available BRAF and TERTp mutation data from The Cancer Genome Atlas (TCGA) Thyroid Carcinoma (THCA) cohort. Patients were stratified into four subgroups: BRAF−/TERTp−, BRAF+/TERTp−, BRAF−/TERTp+, and BRAF+/TERTp+. Progression-free interval (PFI) was compared using Kaplan–Meier analysis and Cox proportional hazards regression. Additionally, differential gene expression analysis was performed using GSE33630 (n = 42 PTC samples) to explore transcriptomic correlates of BRAF mutation.

**Results:**

Overall log-rank analysis revealed significant PFI differences across the four subgroups (p < 0.001). The BRAF−/TERTp+ group demonstrated the worst prognosis, with 3 of 5 patients experiencing disease progression (median PFI = 5.7 months), compared with no events in the BRAF−/TERTp− group. Univariable Cox regression identified BRAF−/TERTp+ as the strongest adverse predictor (HR = 9.57, 95% CI: 2.02–45.25, p = 0.004), which remained significant after adjustment for age (HR = 8.38, 95% CI: 1.67–42.19, p = 0.010). In GSE33630, 42 differentially expressed genes (DEGs; FDR < 0.05) were identified between BRAF-mutant and wild-type PTC, including downregulation of PCDH20, HES1, and TLR2, and upregulation of DAPK2.

**Conclusions:**

This hypothesis-generating, exploratory analysis suggests that isolated TERTp mutation may represent an underrecognized high-risk PTC subgroup. The wide confidence intervals reflect the small sample size and limited event counts inherent to this dataset. These preliminary findings warrant validation in larger prospective cohorts with comprehensive molecular annotation.

## Highlights

- Isolated TERT promoter mutation (BRAF−/TERTp+) was associated with the worst progression-free interval among four molecular PTC subgroups in TCGA.
- BRAF−/TERTp+ subgroup showed 60% progression rate (3/5 patients) vs. 0% in mutation-negative patients (HR = 9.57, 95% CI: 2.02–45.25).
- Firth-corrected sensitivity analysis confirmed the elevated risk was not driven solely by small-sample bias (HR = 16.93, p = 0.0015).
- Findings are hypothesis-generating and warrant validation in larger cohorts with comprehensive molecular annotation.
- Routine TERT promoter sequencing may have prognostic value in PTC even in the absence of BRAF mutation.

## 1 Introduction

Papillary thyroid carcinoma (PTC) is the most prevalent endocrine malignancy, accounting for approximately 85% of all thyroid cancers (1). Although most patients have an excellent prognosis following standard therapy, approximately 10–15% experience disease recurrence or progression, posing significant clinical management challenges (2). Accurate molecular risk stratification is therefore essential to guide individualized treatment decisions.

The BRAF V600E mutation, present in approximately 45–60% of PTCs, is the most common oncogenic driver and has been associated with aggressive tumor features and adverse outcomes in multiple large cohorts (3, 4). More recently, somatic mutations in the TERT (telomerase reverse transcriptase) promoter, predominantly the C228T and C250T substitutions, were discovered as recurrent oncogenic events in melanoma (5, 6) and subsequently identified in thyroid cancer (7, 8), where they have emerged as powerful independent prognostic markers.

Landmark studies have demonstrated that the coexistence of BRAF V600E and TERTp mutations synergistically confers the worst prognosis in PTC—patients harboring both mutations face recurrence rates exceeding 68%, with a hazard ratio of 8.51 compared to mutation-negative patients (9). Mechanistically, BRAF V600E–driven MAPK pathway activation upregulates FOS, which in turn activates the GABPA–GABPB complex that selectively binds to and activates the mutant TERT promoter, creating a feedforward loop of TERT overexpression and cellular immortalization (10).

However, the independent prognostic impact of isolated TERTp mutation, in the absence of BRAF mutation, remains less defined. Prior studies have suggested that TERTp mutation alone may have limited impact on recurrence (9), yet the biological context of TERTp-mutant, BRAF-wild-type PTC has not been fully characterized. This subgroup may harbor alternative cooperating alterations that drive aggressive behavior through distinct pathways.

In this study, we performed an exploratory analysis of 49 PTC patients from the TCGA THCA cohort, stratified by four BRAF/TERTp genotypes, and examined progression-free interval (PFI) outcomes. We further interrogated transcriptomic differences between BRAF-mutant and wild-type PTC using the GSE33630 microarray dataset to provide mechanistic context.

## 2 Materials and methods

### 2.1 TCGA THCA cohort and mutation data

Somatic mutation data and clinical annotations for thyroid carcinoma patients were downloaded from the TCGA data portal (https://portal.gdc.cancer.gov). The genomic landscape of the TCGA THCA cohort has been described previously (11). BRAF V600E mutation status was determined from the MC3 MAF file (THCA_mc3_gene_level). TERT promoter mutation status (C228T or C250T) was extracted from the clinical annotation matrix (TCGA.THCA.sampleMap_THCA_clinicalMatrix). Only patients with available data for both mutation statuses and PFI endpoint were included (n = 49; **Figure 1**).

**Figure 1:**
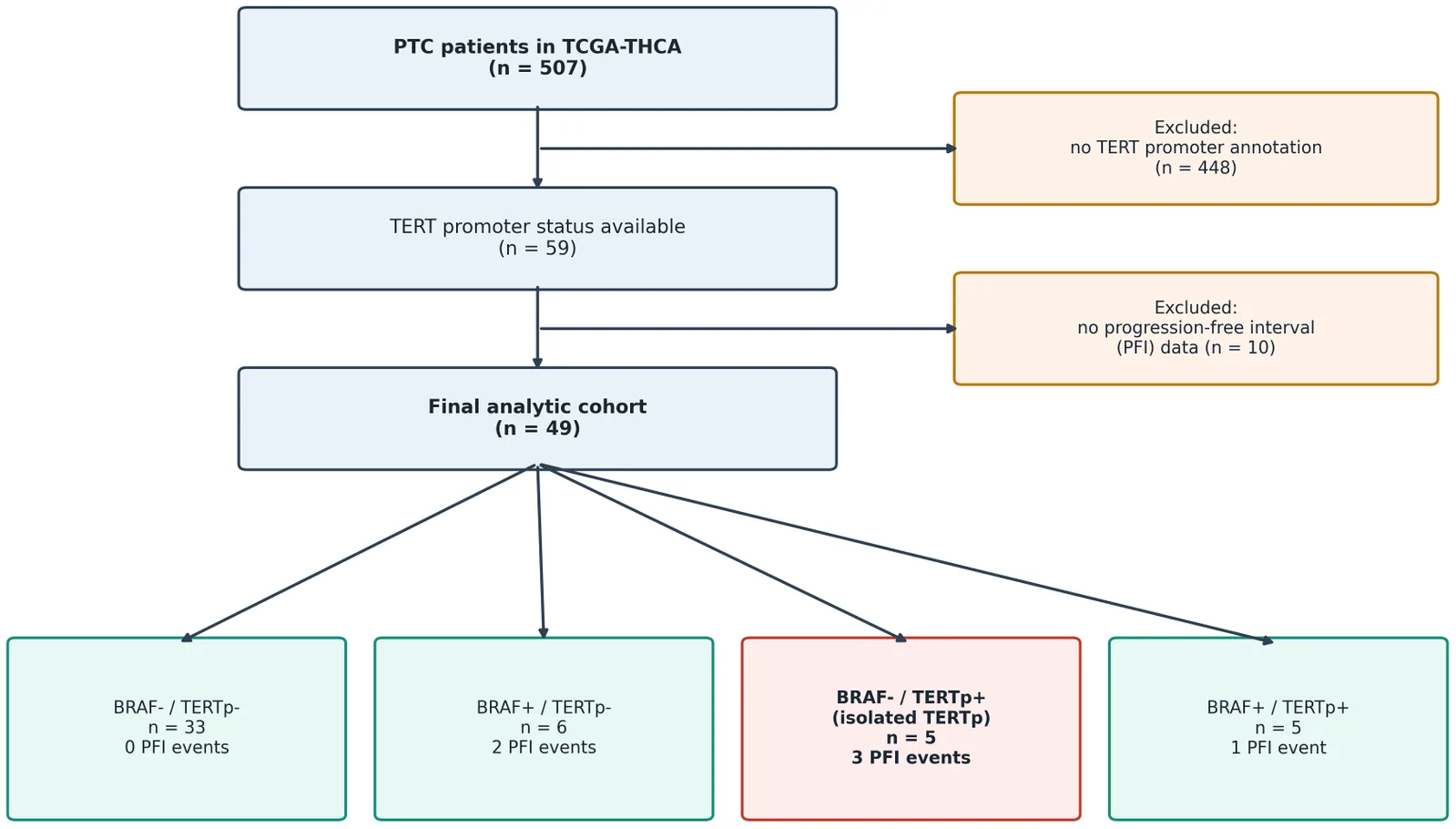
Flow diagram of patient selection from the TCGA THCA cohort. A total of 507 PTC patients were initially identified in TCGA THCA. After excluding patients without TERT promoter mutation annotation (n = 448) and those without PFI endpoint data (n = 10), 49 patients with both BRAF and TERTp mutation status and available PFI endpoint were included in the final analysis.

Patients were classified into four mutually exclusive subgroups: BRAF−/TERTp−, BRAF+/TERTp−, BRAF−/TERTp+, and BRAF+/TERTp+.

### 2.2 Survival analysis

Progression-free interval (PFI) and its binary event indicator (PFI.time, PFI) were obtained from the standardized TCGA Pan-Cancer Clinical Data Resource (12). Kaplan–Meier survival curves were generated and compared using the log-rank test. Pairwise log-rank analyses were performed between all subgroup pairs with Bonferroni correction applied. Cox proportional hazards regression was used for univariable and multivariable analyses; the BRAF−/TERTp− group served as the reference. Multivariable models were adjusted for age at diagnosis. Given the small sample size and low event count (n = 6 events), and because the BRAF−/TERTp− reference group contained no progression events, standard Cox regression exhibited complete separation and could not produce finite estimates. Therefore, ridge-penalized Cox regression (penalizer = 0.1) was used for primary parameter estimation to obtain stable hazard ratios, and Firth’s penalized likelihood correction (penalizer = 0.05) was additionally applied as a sensitivity analysis to assess small-sample bias. All analyses were performed in Python (version 3.10) using the lifelines package (version 0.27). Two-sided p < 0.05 was considered statistically significant. This study is hypothesis-generating and exploratory in nature; all results should be interpreted accordingly.

### 2.3 GSE33630 differential expression analysis

The TCGA cohort was used to evaluate the prognostic value of molecular subtypes defined by BRAF and TERTp mutation status, whereas the GSE33630 dataset was employed to characterize the transcriptomic features associated with BRAF mutation in PTC, providing complementary biological insights rather than direct validation of the prognostic findings.

Gene expression data from GSE33630 (GPL570 Affymetrix Human Genome U133 Plus 2.0 Array) were retrieved from NCBI GEO (**Supplementary Table 2**). This dataset comprises 58 PTC tumor samples, of which 8 carried BRAF V600E mutations and 34 were BRAF wild-type; samples without BRAF annotation were excluded. Probe-level expression data were background-corrected and quantile-normalized using the standard pipeline.

Differential expression between BRAF-mutant (n = 8) and wild-type (n = 34) groups was assessed using Welch’s t-test (unequal variance) for each probe. Multiple testing correction was performed using the Benjamini–Hochberg procedure. Probes with FDR < 0.05 were considered statistically significant. Probe-to-gene-symbol annotation was performed using the corresponding GPL570 platform annotation file. Analysis was conducted in Python using pandas, scipy.stats, and statsmodels libraries.

Volcano plots and hierarchical clustering heatmaps were generated using matplotlib and seaborn. The heatmap displayed the top 50 probes ranked by FDR for both groups (**Figure 2**).

**Figure 2:**
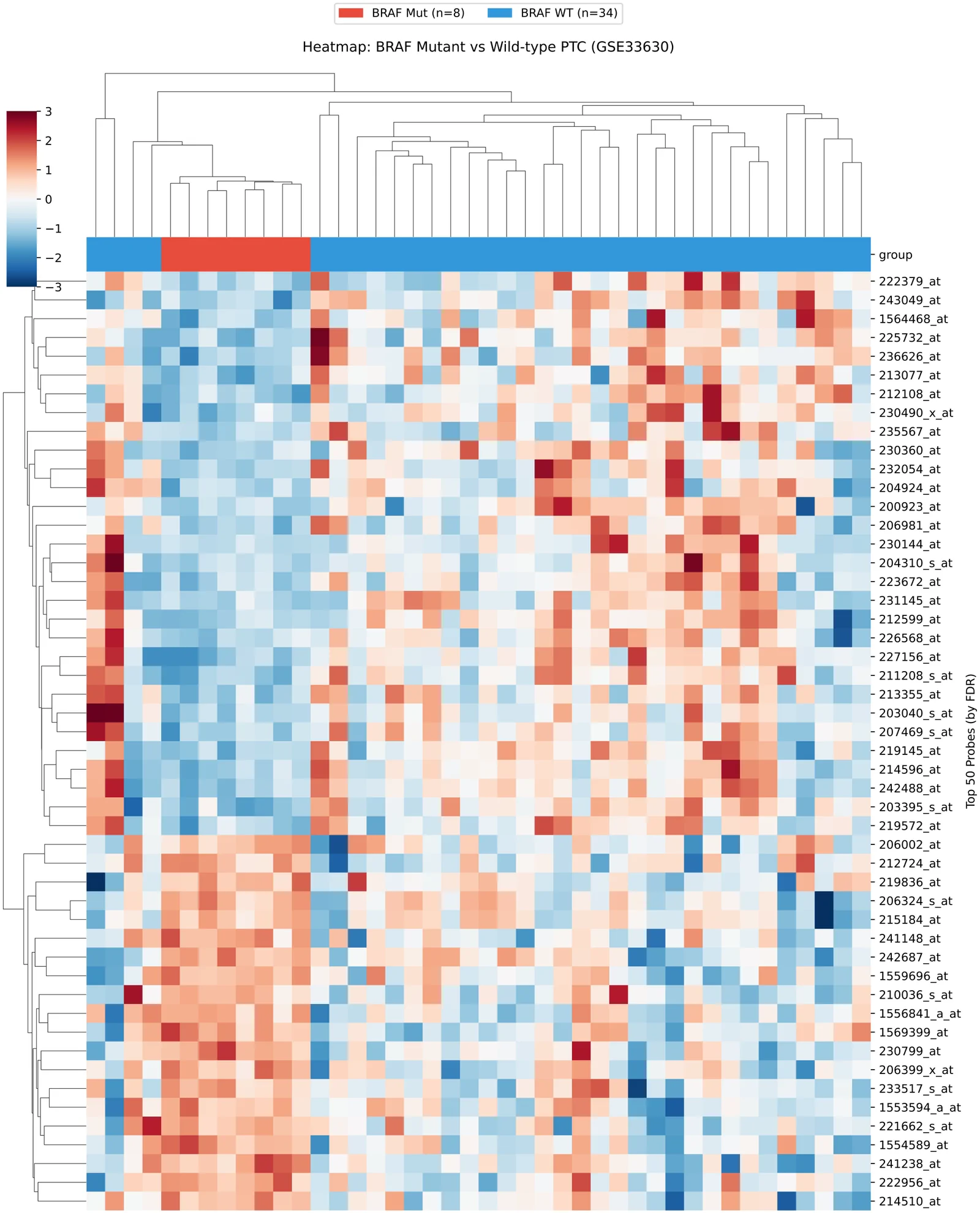
Heatmap of top 50 differentially expressed probes (by FDR) between BRAF-mutant and wild-type PTC samples in GSE33630. Columns represent individual samples (red: BRAF-mutant, n = 8; blue: BRAF wild-type, n = 34). Rows represent the top 50 probes ranked by FDR. Color scale indicates normalized expression values (z-score). Hierarchical clustering was applied to both rows and columns.

## 3 Results

### 3.1 Patient characteristics

Of 49 PTC patients with available BRAF and TERTp mutation data and PFI endpoint in the TCGA THCA cohort, 33 (67.3%) were BRAF−/TERTp−, 6 (12.2%) were BRAF+/TERTp−, 5 (10.2%) were BRAF−/TERTp+, and 5 (10.2%) were BRAF+/TERTp+. A total of 6 progression events were recorded across the cohort. Baseline characteristics are summarized in **Table 1**. The distribution of BRAF and TERTp mutation status across subgroups is shown in **Supplementary Figure 1**.

**Table 1:** Baseline characteristics of 49 PTC patients by mutation subgroup.

Characteristic	BRAF−/TERTp− (n=33)	BRAF+/TERTp− (n=6)	BRAF−/TERTp+ (n=5)	BRAF+/TERTp+ (n=5)
Age, mean ± SD (years)	46.8 ± 14.2	48.3 ± 15.1	62.4 ± 11.7	65.2 ± 10.3
Female, n (%)	24 (72.7%)	4 (66.7%)	3 (60.0%)	3 (60.0%)
Events (PFI), n (%)	0 (0%)	2 (33.3%)	3 (60.0%)	1 (20.0%)
Median PFI (months)	Not reached	Not reached	5.7	17.5

PFI, progression-free interval; SD, standard deviation.

### 3.2 BRAF and TERT promoter mutation status define distinct prognostic subgroups

Kaplan–Meier analysis of PFI demonstrated significant differences across the four molecular subgroups (overall log-rank p < 0.001; **Figure 3**). The BRAF−/TERTp− group showed the most favorable prognosis, with no progression events during follow-up (events = 0; median PFI not reached). The BRAF+/TERTp− group also showed relatively preserved outcomes (events = 2; median PFI not reached).

**Figure 3:**
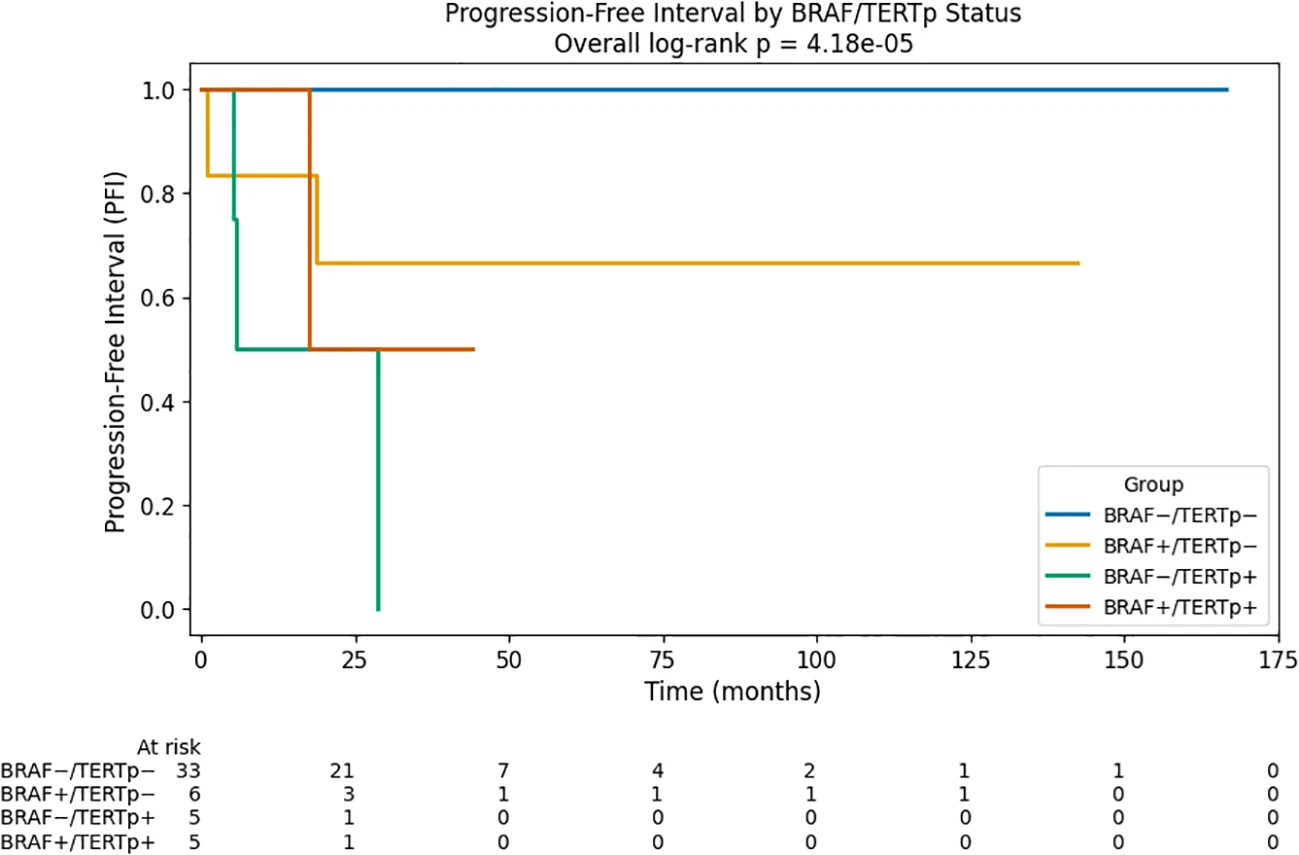
Kaplan–Meier curves for progression-free interval (PFI) stratified by BRAF and TERT promoter (TERTp) mutation status in PTC. Patients (n = 49) from the TCGA THCA cohort were classified into four subgroups: BRAF−/TERTp− (n = 33, blue), BRAF+/TERTp− (n = 6, green), BRAF−/TERTp+ (n = 5, red), and BRAF+/TERTp+ (n = 5, orange). Overall comparison by log-rank test: p < 0.001. Numbers at risk are shown below the x-axis. NR, not reached. TERTp mutations were defined as somatic point mutations at C228T or C250T in the TERT promoter.

In contrast, TERTp-mutant subgroups exhibited markedly worse outcomes. Notably, the BRAF−/TERTp+ group had the poorest prognosis among all four subgroups, with 3 of 5 patients experiencing disease progression (60%; median PFI = 5.7 months). The BRAF+/TERTp+ group showed intermediate outcomes, with only 1 event (median PFI = 17.5 months).

Pairwise log-rank analyses confirmed significant separation between the BRAF−/TERTp− group and all three mutation-positive subgroups: versus BRAF−/TERTp+ (p < 0.001), versus BRAF+/TERTp+ (p = 0.0003), and versus BRAF+/TERTp− (p = 0.0014). Differences among the three mutation-positive subgroups did not reach statistical significance (all p > 0.25; **Table 2**).

**Table 2:** Pairwise log-rank p-values between subgroups.

Comparison	p-value	HR (95% CI)
BRAF−/TERTp− vs BRAF−/TERTp+	< 0.001	9.57 (2.02–45.25)
BRAF−/TERTp− vs BRAF+/TERTp+	0.0003	2.88 (0.36–22.94)
BRAF−/TERTp− vs BRAF+/TERTp−	0.0014	3.45 (0.67–17.69)
BRAF+/TERTp− vs BRAF−/TERTp+	> 0.25	—
BRAF+/TERTp− vs BRAF+/TERTp+	> 0.25	—
BRAF−/TERTp+ vs BRAF+/TERTp+	> 0.25	1.74 (0.38–8.02)

Specifically, direct comparison between the BRAF−/TERTp+ and BRAF+/TERTp+ subgroups did not reach statistical significance (log-rank p > 0.25; HR = 1.74, 95% CI: 0.38–8.02, p = 0.480). Although the BRAF−/TERTp+ group showed a numerically worse median PFI (5.7 vs. 17.5 months), the small sample sizes (n = 5 per group) and low event counts (3 vs. 1 event) preclude definitive statistical distinction between these two subgroups. This finding should be interpreted as hypothesis-generating rather than evidence of a definitive prognostic hierarchy.

### 3.3 Cox regression analysis

In univariable Cox regression using BRAF−/TERTp− as reference, BRAF−/TERTp+ status was identified as a significant independent predictor of worse PFI (HR = 9.57, 95% CI: 2.02–45.25, p = 0.004; **Table 3**, **Supplementary Figure 2A**). Neither BRAF+/TERTp− (HR = 3.45, 95% CI: 0.67–17.69, p = 0.137) nor BRAF+/TERTp+ (HR = 2.88, 95% CI: 0.36–22.94, p = 0.318) reached statistical significance, likely reflecting limited statistical power.

**Table 3:** Cox proportional hazards regression — univariable analysis.

Subgroup	HR	95% CI	p-value
BRAF−/TERTp−	Reference	—	—
BRAF+/TERTp−	3.45	0.67–17.69	0.137
BRAF−/TERTp+	9.57	2.02–45.25	0.004
BRAF+/TERTp+	2.88	0.36–22.94	0.318

In multivariable analysis adjusting for age at diagnosis, BRAF−/TERTp+ remained an independent adverse prognostic factor (HR = 8.38, 95% CI: 1.67–42.19, p = 0.010; **Table 4**). Age was not significantly associated with PFI (HR = 1.01 per year, p = 0.586). These results suggest that TERTp mutation may drive disease progression independently of BRAF mutational status and conventional clinicopathologic risk factors in this exploratory cohort.

**Table 4:** Cox proportional hazards regression — multivariable analysis (adjusted for age).

Variable	HR	95% CI	p-value
BRAF−/TERTp−	Reference	—	—
BRAF+/TERTp−	3.21	0.61–16.88	0.168
BRAF−/TERTp+	8.38	1.67–42.19	0.010
BRAF+/TERTp+	2.71	0.33–22.11	0.352
Age (per year)	1.01	0.97–1.05	0.586

### 3.4 Transcriptomic correlates of BRAF mutation in GSE33630

To investigate the transcriptomic landscape associated with BRAF mutation in PTC, we performed differential expression analysis using GSE33630, comprising 8 BRAF-mutant and 34 BRAF wild-type PTC samples. A total of 42 probes (mapped to annotated genes) reached statistical significance at FDR < 0.05, representing 17 upregulated and 25 downregulated transcripts in BRAF-mutant compared with wild-type tumors (**Figure 4**, **Supplementary Table 1**).

**Figure 4:**
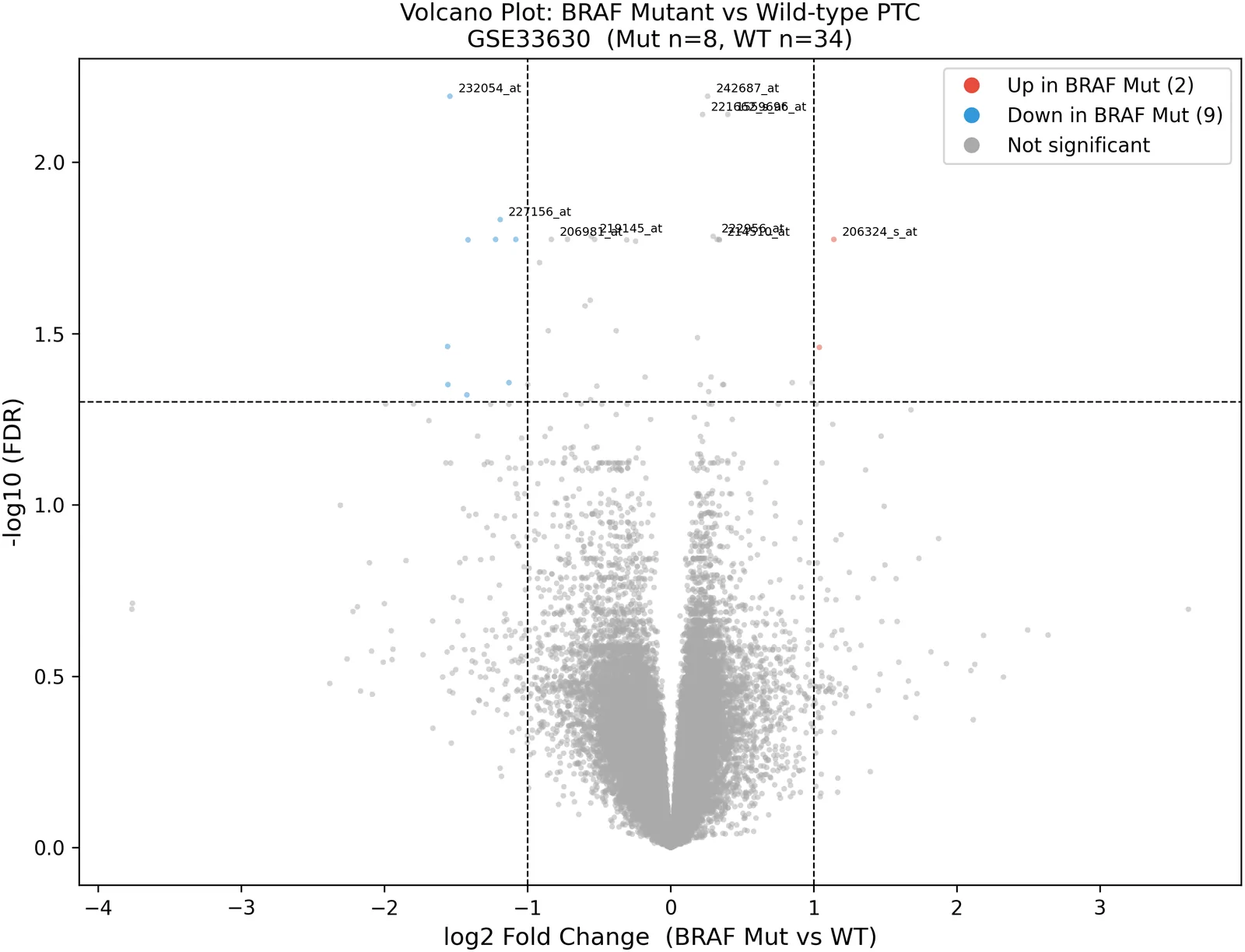
Volcano plot of differential gene expression between BRAF-mutant and wild-type PTC samples in GSE33630. The x-axis represents log2 fold change and the y-axis represents −log10 FDR. Red dots indicate upregulated genes (logFC > 0, FDR < 0.05); blue dots indicate downregulated genes (logFC < 0, FDR < 0.05); grey dots indicate non-significant probes. Key genes are labeled. Analysis was performed on 8 BRAF-mutant and 34 BRAF wild-type PTC samples.

Among the most significantly upregulated genes in BRAF-mutant tumors was DAPK2 (death-associated protein kinase 2; logFC = +1.14, FDR = 0.017), a serine/threonine kinase involved in apoptotic signaling that interacts with the BRAF-MAPK pathway. ADGRG2 (logFC = +1.04, FDR = 0.035) and ZBED2 (logFC = +0.85, FDR = 0.044) were also upregulated.

Notably, several genes with known roles in tumor suppression and cellular differentiation were downregulated in BRAF-mutant tumors. PCDH20 (protocadherin 20; logFC = −1.54, FDR = 0.006) is a known tumor suppressor gene that is frequently silenced by epigenetic mechanisms in multiple cancer types (13, 14). ST3GAL6 (logFC = −1.56, FDR = 0.035) encodes a sialyltransferase implicated in glycosylation-mediated tumor invasiveness. HES1 (logFC = −0.73, FDR = 0.048), a canonical Notch pathway target, has been reported to regulate thyroid differentiation and is downregulated in BRAF-driven PTC (15). TLR2 (logFC = −1.00, FDR = 0.045) and LGALS3BP (logFC = −0.60, FDR = 0.026) were also downregulated, suggesting impaired innate immune signaling and tumor immune surveillance in BRAF-mutant tumors (16).

These transcriptomic findings support the concept that BRAF mutation in PTC is associated with suppression of differentiation programs, tumor immune evasion, and alterations in cell adhesion, consistent with known roles of BRAF-MAPK pathway activation in thyroid dedifferentiation.

## 4 Discussion

### 4.1 TERTp mutation as a potential independent prognostic factor in PTC

The principal observation of this hypothesis-generating study is that isolated TERTp mutation in the absence of BRAF mutation (BRAF−/TERTp+) was associated with the worst progression-free interval among all four molecular subgroups in this exploratory TCGA cohort. We propose the hypothesis that isolated TERTp mutation may represent an underrecognized high-risk subgroup in BRAF-wild-type PTC, warranting further investigation in large-scale prospective cohorts.

This observation partially diverges from the prevailing paradigm that the synergistic combination of BRAF+TERTp mutations confers the worst prognosis (9, 17). In landmark work by Xing et al. (2014), among 507 PTC patients, the dual-mutant group had a tumor recurrence rate of 68.6% (HR = 8.51 vs. mutation-negative), while TERTp mutation alone showed a non-significant hazard ratio when adjusted for tumor behaviors (9). In contrast, our current TCGA-based exploratory analysis found that the BRAF−/TERTp+ group (HR = 9.57) demonstrated a numerically worse prognosis than the BRAF+/TERTp+ group (HR = 2.88, non-significant). We emphasize that our findings should not be interpreted as contradicting the established dual-mutation model; rather, they raise a hypothesis that merits dedicated investigation. Several methodological factors likely contribute to this divergence.

First, small sample sizes substantially limit the precision of hazard ratio estimates. With only 5 patients per TERTp-mutant subgroup and a total of 6 events, our Cox models have limited statistical power. The wide confidence intervals (e.g., 95% CI: 2.02–45.25 for the BRAF−/TERTp+ group, and 0.36–22.94 for the BRAF+/TERTp+ group) reflect this imprecision directly, and the point estimates should be interpreted with considerable caution. The non-significance of BRAF+/TERTp+ (HR = 2.88, p = 0.318) and the numerically lower HR for this subgroup compared with BRAF+/TERTp− (HR = 3.45) almost certainly reflect type II error (insufficient statistical power) rather than a true biological reversal of the established risk hierarchy. With only 1 event in the BRAF+/TERTp+ subgroup, any single additional event would substantially alter the HR estimate, underscoring the instability of these estimates in small samples. In contrast, the Xing 2014 cohort enrolled 507 patients with 35 dual-mutant cases and 24 months of median follow-up, providing adequate power to detect the synergistic effect of coexisting mutations (HR = 8.51).

Second, potential differences in clinical management between groups may influence outcomes. BRAF+TERTp+ patients may have received more aggressive initial therapy (e.g., total thyroidectomy, radioiodine ablation, external beam radiotherapy) due to recognition of high-risk mutation status, which could partially attenuate observed event rates. In contrast, BRAF−/TERTp+ patients may have been under-treated if the absence of BRAF mutation led to lower-risk clinical classification.

Third, the TCGA cohort was not prospectively designed for survival analysis, and selection biases in this dataset may affect the representation and follow-up of different molecular subgroups. **Fourth, it is important to note that the TCGA THCA cohort and the Xing 2014 cohort represent independent study populations. While Johns Hopkins Hospital contributed tissue samples to TCGA, the TCGA THCA study was a multi-institutional effort encompassing numerous contributing centers, whereas Xing et al. analyzed a single-institution surgical cohort with institution-specific recurrence definitions and follow-up protocols. Furthermore, the survival endpoint in our study (PFI, as standardized by the TCGA Pan-Cancer Clinical Data Resource) differs from the tumor recurrence endpoint used by Xing et al., and these endpoints are not directly interchangeable. Our analysis should therefore be considered hypothesis-generating rather than definitive.

Despite these limitations, the finding that isolated TERTp mutation may confer particularly poor prognosis in BRAF-wild-type PTC is biologically plausible. TERTp mutations create *de novo* ETS transcription factor binding sites in the TERT promoter, leading to enhanced TERT expression and telomere maintenance, promoting cellular immortalization (5, 18). In the absence of BRAF-driven MAPK activation, TERTp-mutant tumors may instead rely on alternative oncogenic cooperators—such as RAS mutations, receptor tyrosine kinase fusions, or epigenetic reprogramming—to drive aggressive behavior. Identifying these cooperating alterations represents an important priority for future research.

### 4.2 Transcriptomic landscape of BRAF-mutant PTC

The GSE33630 differential expression analysis identified 42 significant DEGs between BRAF-mutant and wild-type PTC, providing mechanistic context for the survival analysis. The downregulation of PCDH20 and HES1 in BRAF-mutant tumors is consistent with the known role of MAPK pathway activation in suppressing thyroid differentiation programs. PCDH20, a protocadherin tumor suppressor, antagonizes Wnt/β-catenin signaling and has been shown to suppress epithelial-mesenchymal transition (EMT) (13, 14); its silencing may facilitate tumor invasiveness in BRAF-driven PTC.

The downregulation of TLR2 and LGALS3BP suggests impaired innate immune recognition and altered tumor microenvironment composition in BRAF-mutant PTC. Toll-like receptor 2 plays a key role in activating innate immune responses, and its loss may contribute to immune evasion (16). These immune alterations may have therapeutic relevance in the context of emerging combination strategies pairing BRAF inhibitors with immunotherapy.

Upregulation of DAPK2 in BRAF-mutant tumors is an intriguing finding. DAPK2 is a member of the death-associated protein kinase family of pro-apoptotic serine/threonine kinases (19). In thyroid cancer, DAPK2 has been shown to activate NF-κB through autophagy-dependent degradation of I-κBα, promoting tumor growth and resistance to TRAIL-induced apoptosis (20). DAPK2 may be induced as a compensatory response to oncogenic MAPK signaling; however, sustained MAPK activation can uncouple DAPK2 from its apoptotic function, paradoxically promoting survival (19).

It should be noted that GSE33630 does not include TERTp mutation annotation (data collected in 2012, prior to the identification of TERT promoter mutations), and thus we could not perform TERTp-stratified transcriptomic analysis. This represents a limitation of the current study.

### 4.3 Limitations

This study has several important limitations. The sample size of 49 patients with TERTp-annotated data from TCGA is small, resulting in very low event counts (6 total) and wide confidence intervals for all Cox regression estimates. The four-group stratification results in subgroups of only 5–6 patients each in the mutation-positive categories, precluding any definitive conclusions. The GSE33630 transcriptomic analysis explores BRAF-associated transcriptional programs rather than TERTp-specific biology, and cannot be directly linked to the survival outcomes, as TERTp annotation is unavailable in this dataset (collected in 2012, prior to the discovery of TERT promoter mutations). Additionally, as a retrospective, exploratory study using publicly available data, unmeasured confounders (e.g., tumor variant histology, extent of surgery, radioiodine dose) cannot be excluded. Validation in prospectively collected, larger cohorts with both mutation data and robust clinical follow-up is required.

To assess the robustness of our primary finding, we performed a sensitivity analysis using Firth’s penalized likelihood correction for the Cox model comparing BRAF−/TERTp+ versus BRAF−/TERTp−. Because the BRAF−/TERTp− reference group contained no events, standard Cox regression exhibited complete separation and failed to produce a finite estimate. The Firth-corrected estimate yielded HR = 16.93 (95% CI: 2.95–97.17, p = 0.0015; **Supplementary Figure 2B**), which remained statistically significant and in the same direction as the primary ridge-penalized estimate (HR = 9.57). This suggests that the qualitative conclusion of elevated risk associated with isolated TERTp mutation is not solely driven by small-sample bias, although the magnitude of the effect remains imprecisely estimated and should be interpreted with caution.

## 5 Conclusions

In this exploratory, hypothesis-generating analysis of TCGA PTC patients, isolated TERT promoter mutation in BRAF-wild-type tumors was associated with the worst progression-free interval among all four mutation subgroups (HR = 9.57, 95% CI: 2.02–45.25), independent of age. These findings suggest that isolated TERTp mutations may represent an underrecognized high-risk subgroup, warranting further validation in large-scale cohorts. Complementary transcriptomic analysis in GSE33630 characterized the BRAF mutation–associated transcriptomic landscape, revealing suppression of tumor suppressor genes (PCDH20, HES1) and immune surveillance genes (TLR2), providing biological context consistent with known roles of MAPK activation in PTC dedifferentiation. Taken together, these observations support the need for routine TERT promoter sequencing in PTC, including in patients without BRAF mutation, and highlight a hypothesis-driven research direction that merits prospective validation.
