# Supplementary material for: Isolated TERT promoter mutation may define a high-risk prognostic subgroup in papillary thyroid carcinoma: an exploratory, hypothesis-generating analysis of TCGA and GSE33630 datasets
Source: Front Endocrinol (Lausanne). 2026 Oct 15;17:1881969. doi: 10.3389/fendo.2026.1881969 (PMC13716624; doi:10.3389/fendo.2026.1881969)
Supplement: Supplementary file 1 [file Supplementaryfile1.docx]

# Supplementary Materials

## Isolated TERT Promoter Mutation May Define a High-Risk Prognostic Subgroup in Papillary Thyroid Carcinoma: An Exploratory, Hypothesis-Generating Analysis of TCGA and GSE33630 Datasets

## Supplementary Methods

### S1. TCGA Data Retrieval and Processing

Somatic mutation data were obtained from the TCGA GDC portal. The *BRAF* V600E (p.Val600Glu; c.1799T>A) mutation was identified from the MC3 somatic mutation calling pipeline (THCA_mc3_gene_level.txt). *TERT* promoter mutation status (C228T and/or C250T) was derived from the TCGA THCA clinical annotation matrix, where TERTp mutation was annotated as a binary variable. Patients were included if they had non-missing values for both mutation variables and the PFI endpoint. Patients without annotated *TERT* promoter status were excluded from analysis.

Survival endpoints (PFI and PFI.time, in days) were obtained from the pan-cancer clinical data resource (Liu et al., *Cell*, 2018; PMID: 29625055). PFI.time was converted to months by dividing by 30.44.

### S2. Statistical Methods — Survival Analysis

Kaplan–Meier estimates were generated using the KaplanMeierFitter class from the lifelines Python package (v0.27.0). The log-rank test was used for overall group comparison and for pairwise comparisons between all six subgroup pairs. For pairwise comparisons, reported p-values are uncorrected; Bonferroni-corrected threshold for six comparisons is p < 0.0083.

Cox proportional hazards models were fitted using the CoxPHFitter class from lifelines. Because the reference subgroup (BRAF−/TERTp−) had zero progression events, the data exhibited complete separation and standard Cox regression failed to produce finite estimates. Accordingly, both univariable and multivariable models were fitted using an L2 (ridge) penalization term (λ = 0.1), and a Firth-type penalized correction (λ = 0.05) was additionally performed as a sensitivity analysis to verify the robustness of the primary findings. The proportional hazards assumption was not formally tested given the small event count. All models used BRAF−/TERTp− as the reference group.

Statistical analyses were performed in Python 3.10. Two-sided p < 0.05 was considered statistically significant for primary analyses.

### S3. GSE33630 — Data Retrieval and Preprocessing

The GSE33630 dataset (GEO accession: GSE33630; https://www.ncbi.nlm.nih.gov/geo/query/acc.cgi?acc=GSE33630) was generated on the Affymetrix Human Genome U133 Plus 2.0 Array (GPL570) and comprises 58 PTC and normal thyroid tissue samples. For differential expression analysis, only tumor samples with known *BRAF* mutation status were included (8 *BRAF*-mutant, 34 *BRAF* wild-type); samples annotated as normal thyroid tissue and those without *BRAF* status were excluded.

The expression matrix was downloaded from the series matrix file (GSE33630_series_matrix.txt). Probe-level log2-transformed expression values were used directly, as provided in the pre-processed GEO submission. No additional normalization was applied.

### S4. GSE33630 — Differential Expression Analysis

For each probe, Welch’s two-sample t-test (assuming unequal variances) was applied to compare expression between *BRAF*-mutant (n = 8) and wild-type (n = 34) groups. The resulting p-values were corrected for multiple testing using the Benjamini–Hochberg procedure, yielding adjusted p-values (FDR). Probes with FDR < 0.05 were defined as statistically significant.

Log2 fold change (logFC) was computed as the difference in mean log2 expression between *BRAF*-mutant and *BRAF* wild-type groups (logFC = mean_mutant − mean_wildtype).

Probe-to-gene symbol annotation was performed using the GPL570 platform annotation file downloaded from NCBI GEO. For probes mapping to multiple genes, the first listed gene symbol was retained. Probes without a corresponding gene symbol annotation were excluded from interpretation.

All analyses were performed using the following Python libraries: pandas (v1.5.3), numpy (v1.24.1), scipy.stats (v1.10.1), statsmodels (v0.14.0), matplotlib (v3.7.0), seaborn (v0.12.2).

### S5. Visualization

The volcano plot (Figure 2) displays log2 fold change on the x-axis and −log10 FDR on the y-axis. Significance thresholds are indicated by horizontal (FDR = 0.05) and vertical (|logFC| = 0.5) dashed lines. Points are colored by significance and direction: red = upregulated (FDR < 0.05 and logFC > 0), blue = downregulated (FDR < 0.05 and logFC < 0), grey = not significant. Top genes by FDR are labeled.

The heatmap (Figure 3) displays the top 50 probes ranked by FDR. Expression values were z-score normalized across samples before plotting. Hierarchical clustering (Ward linkage, Euclidean distance) was applied to both rows and columns. Sample group assignments (*BRAF* status) are indicated by column color annotations.

## Supplementary Tables

### Table S1. Complete List of Significant Differentially Expressed Genes (FDR < 0.05) in GSE33630 BRAF-Mutant vs. Wild-Type PTC

| Probe ID | Gene Symbol | logFC | FDR | Direction | Biological Role |
| --- | --- | --- | --- | --- | --- |
| 242687_at | DAPK2 | +1.14 | 0.017 | Up | Apoptosis-related kinase; MAPK-interacting |
| 219361_at | ADGRG2 | +1.04 | 0.035 | Up | Adhesion G protein-coupled receptor |
| 228618_at | ZBED2 | +0.85 | 0.044 | Up | Transcriptional regulation |
| 228430_at | HIF3A | +0.27 | 0.047 | Up | Hypoxia/angiogenesis signaling |
| 232054_at | PCDH20 | −1.54 | 0.006 | Down | Tumor suppressor; Wnt signaling antagonist |
| 225696_at | ST3GAL6 | −1.56 | 0.035 | Down | Glycosylation; tumor invasiveness |
| 215888_at | CHRM3 | −1.22 | 0.017 | Down | Muscarinic acetylcholine receptor |
| 223839_at | CASK | −1.19 | 0.015 | Down | Calcium/calmodulin-dependent kinase |
| 213822_at | SGIP1 | −1.42 | 0.048 | Down | Endocytosis regulation |
| 219197_at | PIR | −1.56 | 0.045 | Down | Iron regulation |
| 209760_at | GRIA3 | −1.13 | 0.044 | Down | Glutamate receptor |
| 225655_at | HMBS | −0.92 | 0.020 | Down | Heme biosynthesis |
| 203535_at | TLR2 | −1.00 | 0.045 | Down | Innate immunity; tumor microenvironment |
| 219069_at | LGALS3BP | −0.60 | 0.026 | Down | Immune evasion; galectin-binding protein |
| 209773_at | HES1 | −0.73 | 0.048 | Down | Notch pathway target; differentiation marker |
| *(additional 27 probes)* | *…* | *…* | *…* | *…* | *…* |

*Note: Only selected genes with known biological significance in PTC are shown in the main table. The complete probe list (42 probes) with logFC, p-value, and FDR values is available in the accompanying CSV file (GSE33630_DEG_significant.csv).*

### Table S2. GSE33630 Dataset Overview

| Parameter | Value |
| --- | --- |
| GEO Accession | GSE33630 |
| Platform | GPL570 (Affymetrix U133 Plus 2.0) |
| Total samples | 58 |
| BRAF-mutant tumors | 8 |
| BRAF wild-type tumors | 34 |
| Excluded (normal/unannotated) | 16 |
| Original | publicationGiordano et al., Oncotarget, 2012 |
| Reference | PMID23109752 |

## Supplementary Figures

**Figure S1. Distribution of *BRAF* and *TERTp* mutation status in the TCGA THCA cohort subset (n=49).** Stacked bar chart showing the proportion of patients in each mutation subgroup.

**Figure S2A.** Forest plot of hazard ratios from univariable ridge-penalized Cox regression analysis (penalty λ = 0.1). BRAF−/TERTp− served as the reference group. Points indicate HR estimates; horizontal lines indicate 95% confidence intervals. Dotted vertical line at HR = 1.0.

**Figure S2B.** Forest plot of hazard ratios from univariable Cox regression with Firth-type penalized correction (penalty λ = 0.05), performed as a sensitivity analysis. BRAF−/TERTp− served as the reference group. Points indicate HR estimates; horizontal lines indicate 95% confidence intervals. Dotted vertical line at HR = 1.0.

## Source Data Availability

| Data | Source | Access |
| --- | --- | --- |
| TCGA THCA mutation data | GDC Portal | https://portal.gdc.cancer.gov |
| TCGA survival endpoints | Liu et al. 2018 Cell (Supplementary) | PMID: 29625055 |
| TCGA clinical matrix | UCSC Xena | https://xenabrowser.net |
| GSE33630 expression data | NCBI GEO | https://www.ncbi.nlm.nih.gov/geo/query/acc.cgi?acc=GSE33630 |
| GSE33630 clinical annotation | NCBI GEO Supplementary | GSE33630_clinical-annotation.txt |

## Software and Code Availability

All analysis code is available upon request. Key software packages used:

| Package | Version | Purpose |
| --- | --- | --- |
| Python | 3.10 | Primary analysis environment |
| lifelines | 0.27.0 | Survival analysis (KM, Cox) |
| pandas | 1.5.3 | Data manipulation |
| scipy | 1.10.1 | Statistical tests (Welch’s t-test) |
| statsmodels | 0.14.0 | Multiple testing correction (BH) |
| matplotlib | 3.7.0 | Figure generation |
| seaborn | 0.12.2 | Heatmap visualization |
| numpy | 1.24.1 | Numerical computation |
